# Are Cross-Sectional Imaging Modalities Enough for Sarcopenia Assessment?

**DOI:** 10.5152/tjg.2024.23425

**Published:** 2024-01-01

**Authors:** Haluk Tarık Kani, Elif Tufan

**Affiliations:** 1 Department of Gastroenterology, Marmara University School of Medicine, İstanbul, Turkey; 2 Marmara University School of Medicine, İstanbul, Turkey

Dear Editor,

We have read the recently published article by Cankurtaran et al^1^ with great interest. Crohn’s disease (CD) is a chronic inflammatory condition of the gastrointestinal tract. It is caused by impaired immune response and can have extraintestinal complications. Some clinical factors could be associated with the prognosis of CD in disease outcome.^2,3^

The authors diagnosed sarcopenia by magnetic resonance enterography (MRE) and investigated the relationship of sarcopenia with clinical outcomes in patients with CD. The need for hospitalization, abscess development, the need for surgery, the need for steroids, the first prescription of biological agents, and switching or dose optimization of biologics were all defined as prognostic variables in the study. As one of the main points, the authors indicated that sarcopenia is associated with poor outcomes such as hospitalization, abscess, and surgery. However, no statistically significant difference in hospitalization was seen in relation to sarcopenia. In addition, there was only 1 patient who developed abscess, and 2 patients who did not have sarcopenia underwent surgery. Despite the result being statistically significant, a larger sample size was required to identify sarcopenia as a risk factor for abscess development and surgery (abscess development: 1 vs. 7 and surgery: 2 vs. 17).

As another contribution, sarcopenia diagnosis is complex and requires a stepwise approach and categorization for different patient groups. Sarcopenia is described as loss of muscle mass and poor muscle function by the European Working Group on Sarcopenia in Older People (EWGSOP). The entity is staging as “pre-sarcopenia, sarcopenia, and severe sarcopenia” demonstrated by the decreased muscle mass, muscle strength and performance, respectively according to the suggestion of EWGSOP. Hence, only muscle mass is not enough to determine sarcopenia, since it does not always correlate with muscle strength.^4^

This study measured and used the skeletal muscle area and skeletal muscle index by MRE to determine the presence of sarcopenia.^1^ Consequently, this approach might have overlooked other indices for skeletal muscle strength and quality of muscle, which are crucial for the diagnosis and detecting the clinical long-term consequences.

The EWGSOP recommends using handgrip strength to measure muscle strength, physical performance tests, and various radiological methods for muscle mass. Other studies also point out the importance of incorporating patient’s nutritional status, muscle strength, extent of weight loss, and daily lifestyle into the comprehensive assessment of prognosis.^5^ This more nuanced approach can contribute to a more accurate detection of sarcopenia when compared with imaging techniques alone. The use of these criteria instead of single cross-sectional imaging techniques to diagnose sarcopenia would be more convenient in clinical practice and also in clinical studies.
